# *PRRG4* Brain-Specific Conditional Knockout Mice Display Autism Spectrum Disorder-Like Behaviors

**DOI:** 10.1186/s12575-025-00280-7

**Published:** 2025-05-16

**Authors:** Luxi Shen, Lan Chen, Yuping Tang, Yeyao Yan, Ting Xiong, Yong Liu, Hongzhi Li, Haihua Gu

**Affiliations:** 1 Department of Internal Neurology, Beijing Friendship Hospital, Capital Medical University, Beijing, 100050 China; 2 Department of Laboratory Medicine, Ganzhou Municipal Hospital, Ganzhou Hospital of Guangdong Provincial People’s Hospital, Ganzhou, 341000 China; 3 Key Laboratory of Laboratory Medicine, Ministry of Education, Wenzhou Key Laboratory of Cancer Pathogenesis and Translation, School of Laboratory Medicine and Life Sciences, Wenzhou Medical University, Chashan University Town, Northern Zhongxin Road, Wenzhou, Zhejiang 325035 China

**Keywords:** *PRRG4*, Brain-Specific Conditional Knockout, *Emx1-Cre* Mice, Autism Spectrum Disorder

## Abstract

**Background:**

Autism spectrum disorder (ASD) is a neurodevelopmental disorder characterized primarily by social deficits and repetitive behaviors. The mechanisms of ASD are complex and are not yet fully understood, although many ASD risk genes and mouse models have been reported. It has been suggested that deletion of *PRRG4* (proline-rich and Gla domain 4) deletion may contribute to autism symptoms in patients with WAGR (Wilms’ tumor, aniridia, gonadoblastoma, mental retardation) syndrome. The mouse model with *PRRG4* gene deletion has not been reported so far. This study investigated whether brain-specific conditional knockout of *PRRG4* induces ASD-like symptoms in mice by crossing the *PRRG4*^fl/fl^ mice with *Emx1-Cre* mice, which express *Cre* in the cerebral cortex and hippocampus.

**Results:**

The *PRRG4* brain-specific knockout (*PRRG4*^fl^/^fl^-*Cre*^+^, *PRRG4*-CKO) mice exhibited social deficits, repetitive behaviors, and anxiety-like symptoms compared to *PRRG4*^fl/fl^ control mice according to the results of various behavioral tests. *PRRG4* knockout led to the increase in total dendritic length, branching, and dendritic spine density in the pyramidal neurons of the cerebral cortex and hippocampus, as well as enhanced levels of synaptic proteins including SYP and PSD95. Immunoprecipitation experiment with PRRG4 antibodies showed dramatic decreased interaction of PRRG4 and MAGI2 proteins in brain tissues from *PRRG4*-CKO mice compared to *PRRG4*^fl/fl^ control mice. GST-RBD pull-down assay showed a significant decrease in RhoA-GTP levels in the cerebral cortex and hippocampus of *PRRG4*-CKO mice.

**Conclusions:**

Brain-specific conditional knockout of the *PRRG4* in mice leads to ASD-like symptoms. PRRG4 protein may regulate dendritic and synaptic development in mice by activating RhoA through interaction with MAGI2. These findings provide evidence for a comprehensive understanding of PRRG4 function in vivo and support the association between *PRRG4* loss and ASD phenotypes observed in WAGR syndrome.

## Introduction

Autism spectrum disorders (ASD) are widely recognized as a series of disorders caused by abnormalities in brain neurodevelopment [1]. The core symptoms of ASD are social deficits and repetitive behaviors [2]. ASD is often associated with comorbidities, such as intellectual disability and anxiety [3]. The global prevalence is approximately 1%, with male patients being 4–5 times more prevalent than females [2]. ASD typically begins to manifest before the age of 3 and may persist throughout life. The pathogenesis of ASD is highly complex [4, 5]. Most research on ASD has focused on genetic mutations, and several risk genes associated with ASD have been identified.

It has been reported that the proline-rich and Gla domain 4 (PRRG4) gene may play a role in the development of autism and neurodevelopment. WAGR (Wilms’ tumor, aniridia, gonadoblastoma, mental retardation) syndrome is a rare chromosomal disorder caused by a deletion of *11p14-p12*, with clinical features including Wilms tumor, aniridia, genitourinary malformations, and intellectual disability. Over 20% of patients have autistic symptoms [6]. It is known that Wilms tumor/genitourinary malformations and aniridia are caused by deletions of the WT1 and PAX6 genes, respectively, but the causes of intellectual disability and autistic symptoms remain unclear [6]. Some literature has speculated that autistic symptoms may be related to deletions of *SLC1A2* at 11p13, *PRRG4* at 11p13, or *BDNF* at 11p14.1 by comparing the different phenotypes of individuals with different fragment deletions in 11p14-p12 (31 cases) [6]. Another literature has found that severe developmental disabilities and autistic behaviors of WAGR syndrome are only shown when *PAX6* or *PRRG4* at 11p13 is deleted, by comparing the different phenotypes of individuals with different fragment deletions in 11p14-p12 (2 cases) [7]. Therefore, *PRRG4* deletion is very likely to be the cause of autistic symptoms in patients with WAGR syndrome.

PRRG4 protein is one of the four members of PRRG family [8]. The PRRG family transmembrane proteins have a characteristic Gla (γ-carboxyglutamic acid) domain in the N-terminal extracellular region [8]. The intracellular region of PRRG4 protein mainly includes an SH3 domain binding motif and two WW domain binding motifs (PY motifs) [8, 9]. The PY motif of PRRG4 can bind to various WW domain-containing proteins, such as membrane-associated guanylate kinase 1 (MAGI1) [10], but the functional significance of the interaction between PRRG4 and MAGI1 is still unclear. PRRG4 and Drosophila Comm are considered as functional homologs [11]. Although the overall amino acid sequence homology between human PRRG4 and Drosophila Comm is low, their WW domain binding motifs are identical. Comm protein can regulate the extension and guidance of neuronal axons in Drosophila embryonic development [11], suggesting that the function of PRRG4 may be also required for embryonic neurodevelopment.

In this study, we investigated whether brain-specific conditional knockout of *PRRG4* induces ASD-like symptoms and explored the underlying mechanisms by generating the *PRRG4* brain-specific conditional knockout (CKO) mouse model.

## Methods

### Generation of *PRRG4* Brain-Specific Conditional Knockout Mice and Housing of the Experimental Mice

*PRRG4*^fl/+^ mice with a C57BL/6 background with LoxP sites flanking exon 3 of *PRRG4* were generated by GemPharmatech Co., Ltd (Nanjing, China) using CRISPR-Cas9 technology. *Emx1-Cre* mice (C57BL/6) were kindly provided by Dr. Jieguang Chen at Wenzhou Medical University. In this study, the *Emx1* promoter was used to drive *Cre* expression in the cerebral cortex and hippocampus. Studies have reported that the *Emx1* promoter specifically drives expression in the cerebral cortex and hippocampus of mice [12]. *PRRG4*^fl/fl^ mice were obtained by mating *PRRG4*^fl/+^ mice with each other. *PRRG4*^fl/+^-*Cre*^+^ mice were obtained by mating *Emx1*-*Cre* mice (i.e., *Cre*^+^ mice) with *PRRG4*^fl/fl^ mice. *PRRG4*^fl/fl^-*Cre*^+^ (*PRRG4* brain-specific conditional knockout, referred to as *PRRG4*-CKO) mice and normal control *PRRG4*^fl/fl^ mice were obtained by mating *PRRG4*^fl/+^*-Cre*^+^ mice with *PRRG4*^fl/fl^ mice. Mice were housed under 12 h light/dark cycle, temperature 20℃−25℃, humidity 40%−60%, with sufficient water and food. At the end of the experiment, all mice were euthanized using CO2 followed by cervical dislocation. All animal experiments were approved by the Animal Care and Use Committee and Ethics Committee of Wenzhou Medical University (wydw2023-0214).

### PCR Genotyping of Mice

Tail specimens were clipped from mice at 7–10 days after birth, DNA was extracted, and genotypes were identified by conventional PCR. To identify whether the *PRRG4* gene carries LoxP sites, a pair of primers F1/R1 was designed upstream and downstream of the 5'LoxP site. To identify Cre^+^ mice, in addition to F1/R1 primers, a pair of primers F2/R2 located upstream and downstream of the Cre gene was designed. Primer sequences from 5' to 3': F1: CTGCCAGGAAAGAAGAGTAGGACA, R1: TTACCCATTCAGCTGTCTCCCCA, F2: ATGTCCAATTTACTGACCG, R2: CGCCGCATAACCAGTGAAAC.

### qPCR Detection of *PRRG4* mRNA Expression in Mouse Brain

Mice at postnatal day 10 were anesthetized by inhalation of 1.5% isoflurane, and the cerebral cortex and hippocampus tissues were immersed in an appropriate amount of RNA Keeper Tissue Stabilizer (Vazyme, Nanjing, China) to inactivate endogenous RNase in the tissues. RNA was extracted after homogenization. The extracted RNA was reversely transcribed into cDNA for qPCR. A pair of qPCR primers was designed upstream and downstream of the third exon of *PRRG4* mRNA. Reaction conditions: 95℃ 10 s, 55℃ 30 s, 95℃ 5 s, 40 cycles. qPCR primer sequences from 5' to 3': PRRG4-F: CCACTTCTGATCGTACTCAGCC, PRRG4-R: GCGGTGCATAAAGATGTTTGCT, GAPDH-F: TGGCCTTCCGTGTTCCTAC, GAPDH-R: GAGTTGCTGTTGAAGTCGCA.

### Immunofluorescence Detection of PRRG4 Protein Level in Mouse Brain

Mice at postnatal day 0 were anesthetized by inhalation of 1.5% isoflurane, perfused with 4% paraformaldehyde solution, and the complete brain tissue was separated and fixed in 4% paraformaldehyde solution at 4℃ overnight. The required parts were cut along the coronal plane with a brain mold, dehydrated with 30% sucrose solution, embedded in OCT embedding agent, snap-frozen in liquid nitrogen, and then transferred to −80℃ for long-term storage. Tissue sections (14 μm) of the cerebral cortex and hippocampus were cut, and after the OCT was completely dried, they were stored at −30℃ for long-term storage. Sections were taken out from −30℃, blocked after antigen retrieval. Primary rabbit anti-PRRG4 antibodies (1:100, cat. no. HPA009040, Sigma, Massachusetts, USA) were added and incubated at 4℃ overnight. Secondary antibody (Alexa Fluor 488 labeled anti-rabbit IgG, Beyotime Biotechnology, Jiangsu, China) was added and incubated at room temperature for 2 h in the dark. Nuclei were stained with DAPI (Beyotime Biotechnology) for 4 min. Anti-fluorescence quenching agent was added to the tissue sections, which were covered by coverslips and imaged under a laser scanning confocal microscope.

### Three-Chamber Test for Assessing Social Ability and Social Novelty of Mice

The three-chamber test device was a rectangular box (length × width × height: 60 × 40 × 30 cm), which was divided into three small chambers by partitions, with 5 × 5 cm openings in the partitions. A mouse restraint cage (diameter 8 cm, height 10 cm) was placed in each of the side chambers. The experiment was divided into three stages: habituation stage, social ability test stage, and social novelty test stage. In the habituation stage, both sides were empty cage E, and experimental mice (2–3 months old, 10–11 mice/group) were placed in the middle chamber and allowed to freely explore the two empty cages for 10 min. In the social ability test stage, a non-littermate wild-type mouse (Stranger1, S1) was placed in a cage on one side, and the other side was still an empty cage E. The experimental mice were allowed to freely explore the cage with the stranger mouse S1 and the empty cage E for 10 min. In the social novelty test stage, another non-littermate wild-type mouse (Stranger2, S2) was placed in the other side cage. The experimental mice were allowed to freely explore the cage with the familiar mouse S1 and the cage with the stranger mouse S2 for 10 min. The movement trajectories of all experimental mice were recorded by a video system, and the time spent by the experimental mice in different chambers and cages during different stages was analyzed using behavioral analysis software. Sniffing, direct contact, or climbing the cage by the mice were all counted as cage contact time.

### Marble Burying Test and Repetitive Behavior Test for Assessing Stereotypic and Repetitive Behavior in Mice

Before the marble burying test, clean corn cob (5 cm thick) bedding was spread evenly in the cage (45 × 30 × 25 cm). Different colored glass marbles (~ 1.6 cm) were randomly placed on top of the bedding. Photos were taken to record the original state of the marbles. Then, 2–3 months old experimental mice (10–11 mice/group) were placed in the cage for 30 min. Photos were taken to record the marble burying by the mice. The number of buried marbles was counted when more than 2/3 of the marble surface area was covered by the bedding.

The repetitive behavior test was to observe the spontaneous stereotypic and repetitive behavior of mice in a familiar environment. Experimental mice (2–3 months old, 10–11 mice/group) were placed in their daily living cages and allowed to acclimate for 15 min. Videos were recorded to assess the repetitive grooming by the experimental mice within 10 min. Grooming behaviors included grasping the face, head, or body with two forelimbs, or licking body parts.

### Elevated Cross Maze Test and Open Field Test for Assessing Anxiety Behavior in Mice

The elevated cross maze device was 60 cm above the ground. The horizontal and vertical lengths of the cross-shaped maze were both 50 cm, representing the open and closed arms, respectively. At the beginning of the experiment, experimental mice (2–3 months old, 10–11 mice/group) were placed into the maze from one side of the closed arm, facing away. The test time was 5 min. The movement of the mice in the maze, and the time and number of entries into different arms were recorded. Finally, the anxiety of the mice was assessed by analyzing the time and number of entries into the open arms using behavioral analysis software.

The open field test was performed in a 40 × 40 × 40 cm experimental box. Experimental mice (2–3 month olds, 10–11 mice/group) were placed in the center of the box, and their movement trajectories were recorded by video for 10 min. Behavioral analysis software was used to analyze the movement distance of the mice to assess their motor ability, and the time spent by the mice in the central area of the open field was mainly analyzed to assess their anxiety status.

### Golgi Staining to Detect Dendritic Morphology of Pyramidal Neurons in Mouse Brain

After anesthetizing mice (3 months old, 3 male mice/group) with 1.5% isoflurane inhalation, complete brain tissue was dissected. A Golgi staining kit (FD Neurotech, Columbia, MD, USA) was used according to the manufacturer’s instructions. The brain tissue was immersed in a mixture of solution A and solution B (1:1) and placed at room temperature in the dark for two weeks. It was then transferred to solution C and placed at room temperature in the dark for 3 days, and placed at −80℃ for at least 1 h. Brain tissue was taken out from −80℃, and 100 µm cerebral cortex and hippocampal sections were cut and air-dried naturally at room temperature in the dark. The sections were placed in a mixture of solution D, solution E, and ddH_2_O (1:1:2) for 10 min for alkalization. After dehydrated with ethanol and permeated with xylene, the sections were covered with coverslips. Microscopic images were taken.

The Neuroanatomy-SNT plugin in Image J software was used to visualize neurons, track neuronal dendrites, and measure the total dendritic length of each pyramidal neuron. The Neuroanatomy-Sholl plugin in Image J software was used to analyze the visualized neurons. Concentric circles with a 10 μm radial increment were drawn with the cell body as the origin. The number of intersections of each pyramidal neuron dendrite on all concentric circles was used as the result of dendritic branching. Image J software was used to count the number of spines on the dendrites, and the Segmented line plugin in Image J software was used to measure the dendritic length. The number of spines per 10 μm dendritic length was used as the dendritic spine density.

### Immunoblotting

After anesthetizing mice (6 male mice/group, 3 months old) with 1.5% isoflurane inhalation, complete fresh brain tissue was dissected, and the cerebral cortex and hippocampal tissues were further separated. Brain tissues were homogenized in RIPA buffer (Beyotime Biotechnology), lysed at 4℃ for 30 min, and centrifuged at 12,000 g for 20 min. The supernatant lysates were analyzed by the BCA method, separated by SDS-PAGE electrophoresis, transferred to PVDF membrane, and immunoblotted with corresponding primary antibodies including anti-PSD95 antibody (1:2000; cat. no. 3450; Cell Signaling Technology), anti-SYP antibody (1:8000; cat. no. A6344; ABclonal Technology, Wuhan, China), anti-MAGI2 antibody (1:5000; cat. no. 25189–1-AP; Proteintech, Wuhan, China), anti-RhoA antibody (1:1000; cat. no. SAB4501661; Sigma), anti-β-actin antibody (1:2000; cat. no. 3700; Cell Signaling Technology), and incubated at 4℃ overnight. Secondary antibodies were added, including HRP-labeled anti-mouse antibody (1:5000; cat. no. A0216; Beyotime Biotechnology) or HRP-labeled anti-rabbit antibody (1:5000; cat. no. A0239; Beyotime Biotechnology), and incubated at room temperature for 1 h. The immunoblots were developed using ECL reagent. Band densitometry analysis was performed using Image Lab software.

### Generation of SH-SY5Y Cells Overexpressing PRRG4

HEK293T17 cells were co-transfected with recombinant lentiviral plasmid pCDH-PRRG4-Flag, or empty vector plasmid pCDH together with the packaging plasmid pSPAX2, and pMD2G. Lentiviral supernatants were harvested 48 h post transfection, and used to infect human neuroblastoma cell line SH-SY5Y purchased from ATCC in the presence of 8 μg/mL polybrene. The medium was changed after 16 h of infection, and 1.0 μg/mL puromycin was added to the culture dish 6–8 h later.

### Preparation and Purification of His-PRRG4 Antigen Protein

IPTG was used to induce host bacteria to express His-tagged mouse PRRG4 antigen protein (aa135-226) by the PET-28a vector: Bacteria culture was induced with IPTG (final concentration 1 mmol/L) at 37℃ for 4 h, and centrifuged at 4℃, 4000 rpm for 10 min. The bacteria pellets were fully resuspended in denaturing lysis buffer containing 8 M urea and 2 mM PMSF and broken by ultrasonic disruption. The resin of the nickel column was mixed with the denatured PRRG4 protein and incubated on a 4℃ rotary shaker overnight. The resin was repeatedly washed with denaturing lysis buffer and eluted with denaturing elution buffer. The eluted denatured PRRG4 protein was renatured by dialysis using PBS.

### Purification of Rabbit Anti-Mouse PRRG4 Polyclonal Antibody

To prepare the PRRG4 affinity column, purified PRRG4 antigen (~ 1 mg) was covalently crosslinked to the SulfoLink™ Coupling Resin (Thermo Fisher, Massachusetts, USA) according to the manufacturer’s instruction. Rabbit sera from rabbits immunized with His-tagged mouse PRRG4 protein (aa135-226) were generated by HUABIO (Hangzhou, China). PRRG4 serum (2 ml) was added to the PRRG4 affinity column and incubated with rotation at 4℃ overnight. The bound PRRG4 antibodies were washed and eluted with 100 mM glycine, pH 2.5.

### Co-Immunoprecipitation

Cell samples were lysed with lysis buffer containing 50 mM Tris, pH 7.4, 150 mM NaCl, 1% Triton X-100, 20 mM beta-glycerol, 10 mM NaF, 2 mM Na_3_VO4, and protease inhibitor cocktail (Bimake, Houston, USA). Fresh cerebral cortex and hippocampal tissue samples from mice (3 male mice/group, 3 months old) were homogenized and lysed with the lysis buffer at 4℃ with rotation for 1 h, and clarified by centrifugation. Purified PRRG4 antibodies (or negative control rabbit antibody IgG) were added at 0.6 µg antibody/1 × 10^6^ cell lysate or 1.0 µg antibody/1 mg tissue lysate and incubated for 2 h, followed by addition of 10 µL protein A agarose bead (50% slurry) and overnight at 4℃. Then, protein A agarose beads were washed with lysis buffer for 5 times, boiled with 1 × SDS loading buffer at 95℃ for 5 min, and detected by immunoblotting with PRRG4 and MAGI2 antibodies.

### GST-RBD Pull-down Assay to Detect Activated RhoA (RhoA-GTP) Content

pGEX-6p-1-GST-RBD plasmid expressing the GST-tagged Rho-binding domain of Rhotekin (GST-RBD) fusion protein was transformed into BL21 host bacteria. The cultured BL21 bacteria were induced with 0.5 mM IPTG for 2 h at 30 °C, pelleted by centrifugation, resuspended in TBS buffer containing 1% Triton X-100, 10 mM DTT, 2 mM PMSF, 300 µg/ml lysozyme, 10 mM MgCl, and 100 µM Dnase I, and sonicated. The broken bacteria lysate was clarified by centrifugation, and was incubated with glutathione (GT) agarose (Ca#16,100, Thermo Fisher) at 4 °C for 90 min. The purified GST-RBD protein on the GT beads were washed with TBS 5 times and stored on ice temporarily. Fresh cerebral cortex and hippocampal tissue samples from mice (3 male mice/group, 3 months old) were homogenized and lysed with buffer B (1% Triton X-100, 0.5% deoxycholate, 200 mM NaCl, 5 mM MgCl2, 10 mM Tris–Cl pH 7.5) (freshly added 2 mM PMSF and 1% protease inhibitors Cocktail) at 4℃ with rotation for 0.5 h. After centrifugation at 4 °C, 12,000 g for 20 min, the supernatant was added to GST-RBD (~ 50 µg) GT beads and incubated at 4 °C with rotation for 1 h. After centrifugation, the GT beads were washed with buffer B five times, and boiled in 100 μL 1 × SDS lysis buffer. GST-PBD pulldowns and input control were analyzed by western blot using RhoA antibody (1:1000, cat. no. SAB4501661, Sigma).

### Statistical Analysis

GraphPad Prism 9.0 software was used for statistical analysis. T-test was used to compare the means between two groups, and ANOVA was used to compare the means between two groups in three or more groups. Variance homogeneity was tested first. If the variance was homogeneous, Tukey was used to calculate the *P* value; if the variance was heterogeneous, Tamhane's T2 was used to calculate the *P* value. *P* < 0.05 indicated a significant difference.

## Results

### Generation of Mice with Brain-Specific Conditional Knockout of *PRRG4* (*PRRG4*-CKO)

**Fig. 1:**
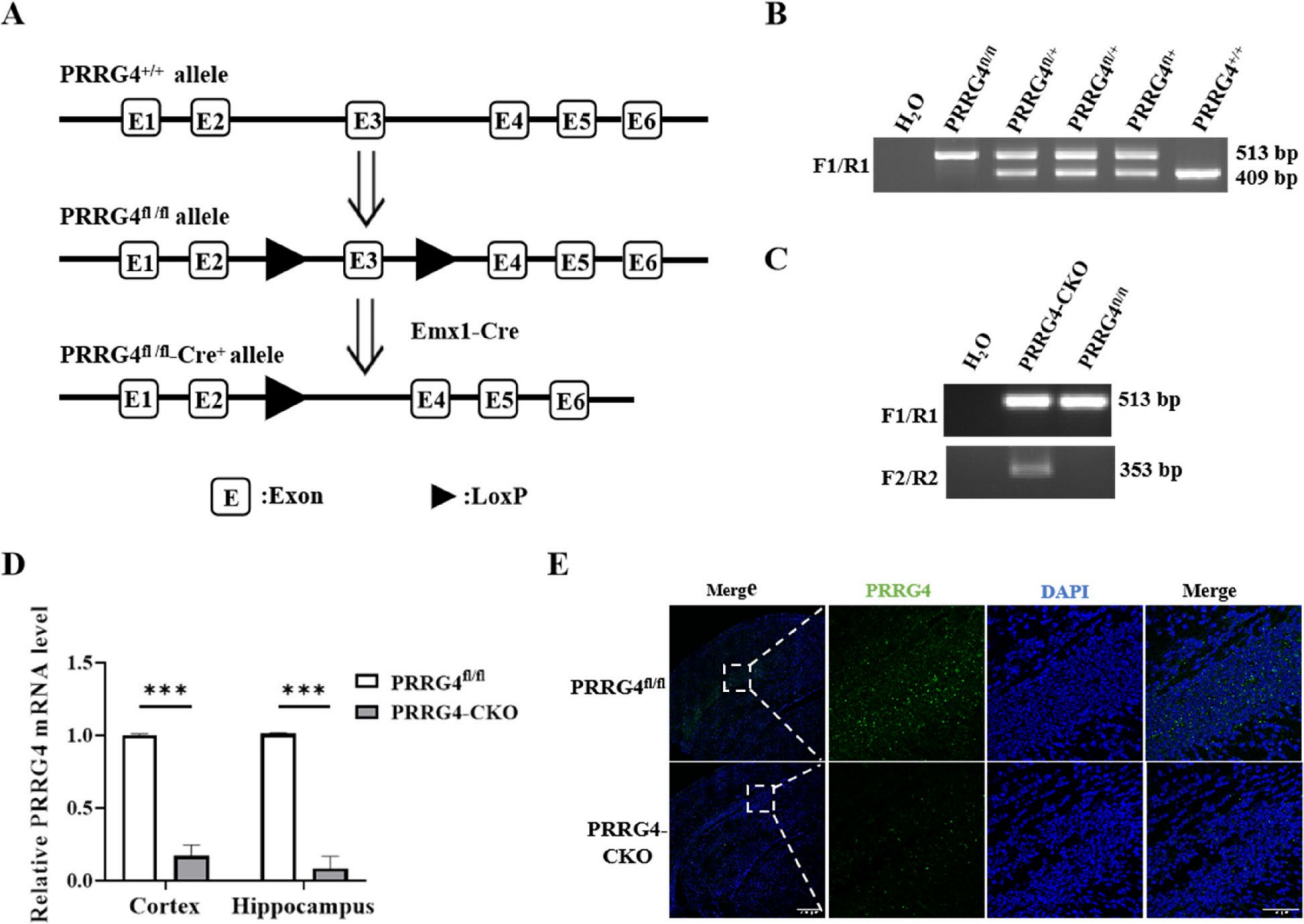
Generation of mice with brain-specific conditional knockout of *PRRG4* (*PRRG4*-CKO). **A** Schematic diagram of knockout *PRRG4* (Exon 3) using *Emx1-Cre*. **B** Genotyping of *PRRG4*^fl/fl^ and *PRRG4*^fl/+^ mice by conventional PCR. **C** Genotyping of *PRRG4*-CKO mice by conventional PCR. **D** qPCR detection of *PRRG4* mRNA levels in the cerebral cortex and hippocampus tissues of *PRRG4*^fl/fl^ and *PRRG4*-CKO mice (3 male mice/group). **E** Immunofluorescence detection of PRRG4 protein levels in the cerebral cortex and hippocampus tissues of *PRRG4*^fl/fl^ and *PRRG4*-CKO mice (scale bar: left first column, 200 μm; second to fourth columns, 50 μm). Note: ***: *p* < 0.001. After generating the *PRRG4*-CKO mice, we assessed various behavioral, morphological, and molecular characteristics to evaluate the impact of *PRRG4* knockout on ASD-like symptoms

To construct *PRRG4* brain-specific conditional knockout mice, we used the Cre-LoxP recombinase system to conditionally knock out the *PRRG4*. The CRISPR/Cas9 gene editing system was used to insert LoxP sites on both sides of exon 3 (E3) of *PRRG4* in the C57BL/6J mouse genome, and then the floxed mice were mated with *Emx1-Cre* mice. The Emx1 promoter drives *Cre* expression specifically in the cerebral cortex and hippocampus of mice [12] (Fig. 1A). F1/R1 PCR primers were used to identify whether *PRRG4* carries the wild type (+) and floxed allele respectively. The results showed that wild-type (*PRRG4*^+/+^) mice only displayed a 409 bp band, *PRRG4*^fl/fl^ mice only exhibited a 513 bp band with the floxed alleles, and *PRRG4*^fl/+^ mice showed the 513 bp and 409 bp bands simultaneously (Fig. 1B). In addition, PCR F2/R2 primers were used to identify the presence of *Cre*^+^ allele in mice, which yielded a 353 bp band (Fig. 1C). Genotyping using both F1/R1 and F2/R2 primers were able to identify the *PRRG4*^fl/fl^-*Cre*^+^ (*PRRG4*-CKO) and *PRRG4*^fl/fl^ control mice, respectively (Fig. 1C).

*PRRG4* knockout in *PRRG4*-CKO mice was further verified by examining the PRRG4 mRNA and protein levels in the cerebral cortex and hippocampus tissues from *PRRG4*^fl/fl^ and *PRRG4*-CKO mice at postnatal day 10. qPCR was used to detect mRNA levels. The results showed that *PRRG4* mRNA was almost undetectable in the cerebral cortex and hippocampus tissues of the *PRRG4*-CKO group, which was significantly lower than the level in the control *PRRG4*^fl/fl^ group (*p* < 0.001) (Fig. 1D). Based on the difference in *PRRG4* mRNA levels in the brain tissues of the *PRRG4*^fl/fl^ and *PRRG4*-CKO groups, the excision efficiency of Cre recombinase was approximately 83% in the cerebral cortex tissue and 92% in the hippocampus tissue (Fig. 1D). Immunofluorescence was used to detect PRRG4 protein levels. The results showed that high levels of PRRG4 fluorescence signals were detected in the cerebral cortex and hippocampus regions of *PRRG4*^fl/fl^ mice, while PRRG4 fluorescence signals were very low in the corresponding regions of *PRRG4*-CKO mice (Fig. 1E). The above results indicated that *PRRG4* was specifically knocked out in the cerebral cortex and hippocampus regions of mice. *PRRG4*-CKO mice were born at the expected mendelian ratio, remained viable, and apparently healthy during the course of this study (data not shown).

### Male and Female *PRRG4*-CKO Mice Exhibit Social Behavior Deficits

**Fig. 2:**
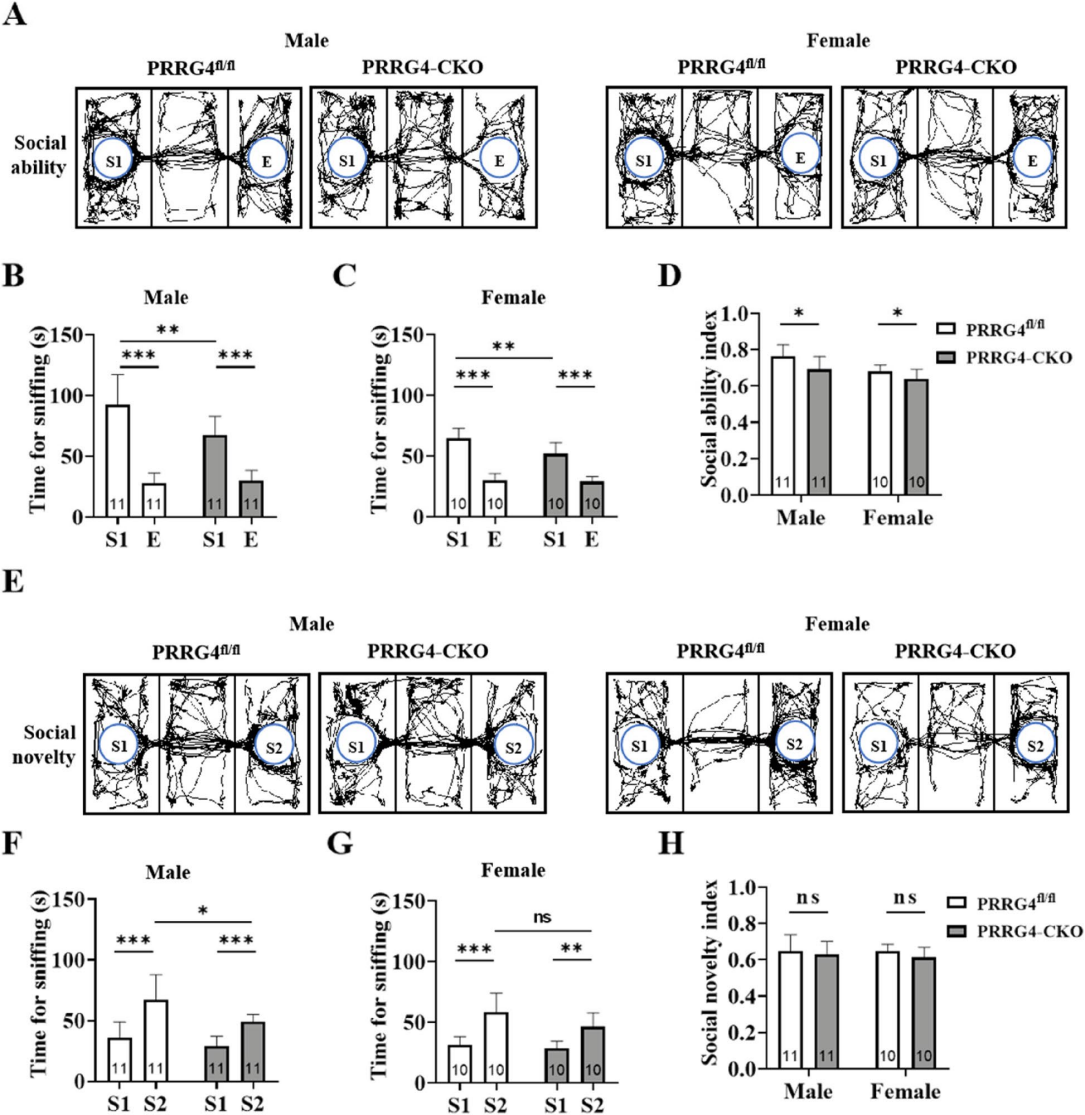
Male and female *PRRG4*-CKO mice exhibit social behavior deficits. **A** Movement trajectory diagrams of male and female *PRRG4*^fl/fl^ and *PRRG4*-CKO mice during the social ability test stage. **B**, **C** Statistical analysis of the time spent contacting the S1 cage and the E cage by male (**B**) and female (**C**) *PRRG4*^fl/fl^ and *PRRG4*-CKO mice during the social ability test stage. **D** Statistical analysis of the social ability index, social ability index = time spent contacting S1 cage/(time spent contacting S1 cage + time spent contacting E cage). **E** Movement trajectory diagrams of male and female *PRRG4*^fl/fl^ and *PRRG4*-CKO mice during the social novelty test stage. **F**, **G** Statistical analysis of the time spent contacting the S1 cage and the S2 cage by male (**F**) and female (**G**) *PRRG4*^fl/fl^ and *PRRG4*-CKO mice during the social novelty test stage. **H** Statistical analysis of the social novelty index, social novelty index = time spent contacting S2 cage/(time spent contacting S2 cage + time spent contacting S1 cage). Note: the numbers of experimental mice in each group (10–11 mice/group) were indicated on the bar. S1 or S2: mouse S1 or S2, E: empty; ***: *p* < 0.001, **: *p* < 0.01, *: *p* < 0.05, ns: no significant statistical difference

Social communication and interaction difficulty is one of the core ASD symptoms. The three-chamber test was used to assess the social ability and novelty of mice. In the habituation stage, the movements of all mice were normal, and there was no significant preference (data not shown). In the social ability testing stage, the movement trajectories of experimental mice contacting the cage with the stranger mouse S1 and the empty cage E were observed (Fig. 2A). The statistical results revealed that both *PRRG4*^fl/fl^ and *PRRG4*-CKO mice, regardless of sex, spent significantly more time contacting the S cage than the E cage (*p* < 0.001) (Fig. 2B, C). However, *PRRG4*-CKO mice, regardless of sex, spent significantly less time contacting the S1 cage than *PRRG4*^fl/fl^ mice (*p* < 0.01) (Fig. 2B, C). In addition, *PRRG4*-CKO mice, regardless of sex, had significantly lower social ability index than *PRRG4*^fl/fl^ mice (*p* < 0.05) (Fig. 2D).

In the social novelty testing stage, the movement trajectories of experimental mice contacting the cage with the stranger mouse S2 and the cage with the familiar mouse S1 were monitored (Fig. 2E). The statistical results showed that both *PRRG4*^fl/fl^ and *PRRG4*-CKO mice, regardless of sex, spent longer time contacting the S2 cage than the S1 cage (*p* < 0.001 or *p* < 0.01) (Fig. 2F, G). However, male *PRRG4*-CKO mice spent significantly less time contacting the S2 cage than male *PRRG4*^fl/fl^ mice (*p* < 0.05) (Fig. 2F). In contrast, there was no significant difference in the time spent contacting the S2 cage between female *PRRG4*-CKO mice and female *PRRG4*^fl/fl^ mice (Fig. 2G). In addition, there was no significant difference in the social novelty index between *PRRG4*-CKO and *PRRG4*^fl/fl^ mice, regardless of sex (Fig. 2H).

### Male and Female *PRRG4*-CKO Mice Display Stereotypic and Repetitive Behaviors

**Fig. 3:**
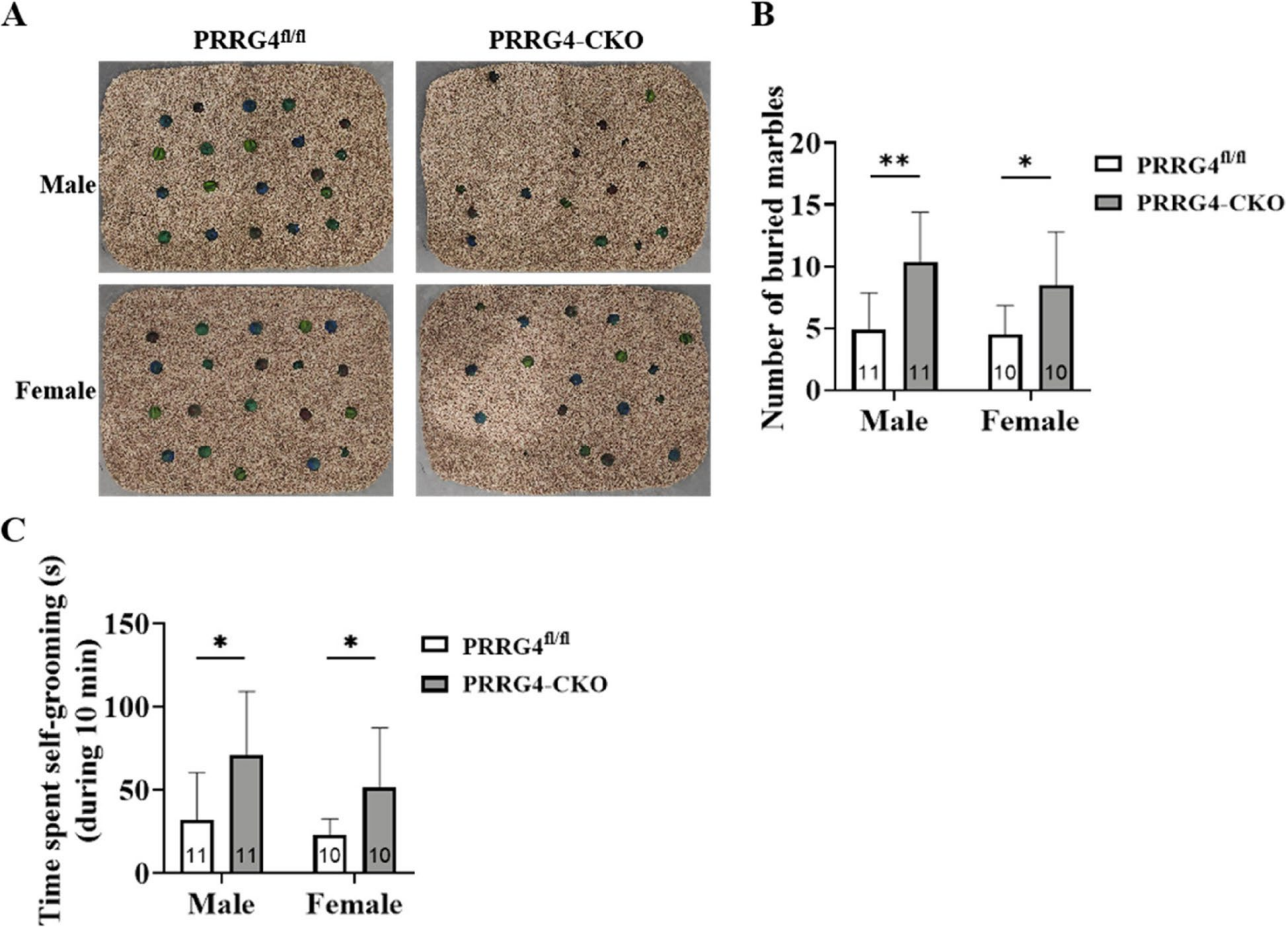
Male and female *PRRG4*-CKO mice display stereotyped repetitive behaviors. **A** Representative images of glass marble buried by male and female *PRRG4*^fl/fl^ and *PRRG4*-CKO mice in the marble burying test. **B** Statistical analysis of the specific number of buried glass marbles. **C** Statistical analysis of the time spent on self-grooming by male and female *PRRG4*^fl/^^fl^ and *PRRG4-*CKO mice. Note: the numbers of experimental mice in each group (10–11 mice/group) were indicated on the bar. **: *p* < 0.01, *: *p* < 0.05

Restricted and repetitive behavior is another core ASD symptom. In the marble burying test, neither male nor female *PRRG4*^fl/fl^ mice showed significant responses to the glass marbles on the bedding surface, and the overall positions of the unburied glass marbles were relatively neat (Fig. 3A). However, both male and female *PRRG4*-CKO mice showed significant responses to the glass marbles, and most of the glass marbles were buried by the mice with bedding (Fig. 3A). The statistical analysis showed that *PRRG4*-CKO mice buried significantly more glass marbles than *PRRG4*^fl/fl^ mice, regardless of sex (*p* < 0.01 or *p* < 0.05) (Fig. 3B).

In the repetitive behavior test, the time spent by the mice on self-grooming within 10 min was used to assess stereotypic and repetitive behaviors. Statistical analysis of the results showed that the average grooming time of *PRRG4*-CKO male mice (70.65 s) was significantly higher than the average grooming time of *PRRG4*^fl/fl^ male mice (31.99 s) (*p* < 0.05) (Fig. 3C). Similarly, the average grooming time of *PRRG4*-CKO female mice (51.33 s) was significantly higher than the average grooming time of *PRRG4*^fl/fl^ female mice (23.18 s) (*p* < 0.05) (Fig. 3C).

### Male *PRRG4*-CKO Mice Manifest Anxiety Symptoms

**Fig. 4:**
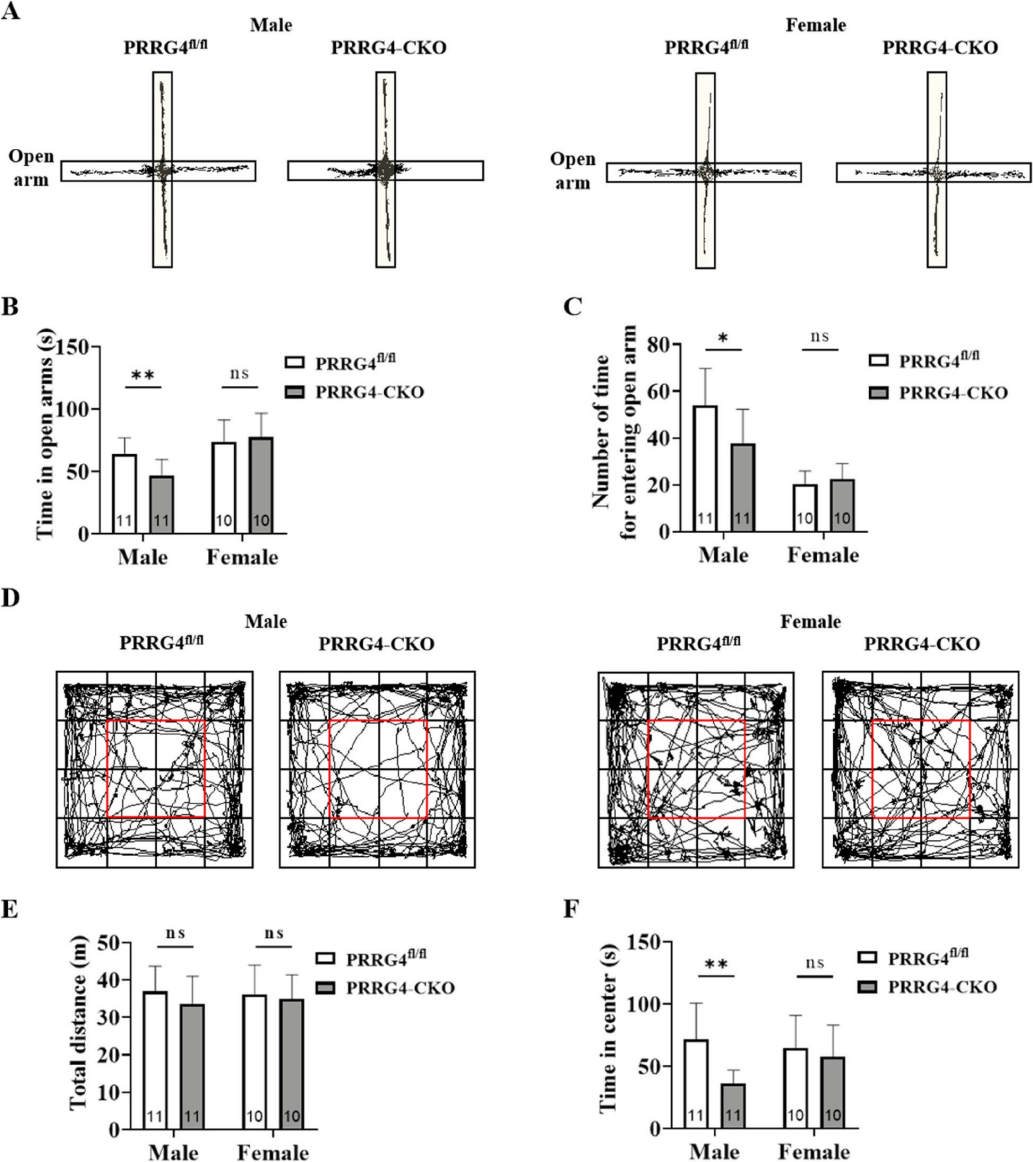
Male *PRRG4*-CKO mice manifest anxiety symptoms. **A** Movement trajectory diagrams of male and female *PRRG4*^fl/fl^ and *PRRG4*-CKO mice in the elevated cross maze test. **B** Statistical analysis of the time spent entering the open arms of the elevated cross maze. **C** Statistical analysis of the number of entries into the open arms of the elevated cross maze. **D** Movement trajectory diagrams of male/female *PRRG4*^fl/fl^ and *PRRG4*-CKO mice in the open field test. **E** Statistical analysis of the total distance traveled in the open field. **F** Statistical analysis of the time spent staying in the central area of the open field. Note: the numbers of experimental mice in each group (10–11 mice/group) were indicated on the bar. **: *p* < 0.01, *: *p* < 0.05, ns: no significant difference

Anxiety is a very common comorbidity in individuals with ASD. In the elevated cross maze test, the movement trajectories of mice in the open and closed arms were recorded to assess anxiety levels (Fig. 4A). Anxious mice have a fear of height and do not like to enter the elevated open arms. The statistical results showed that male *PRRG4*-CKO mice spent significantly less time with less entries into the open arms than male *PRRG4*^fl/fl^ mice (*p* < 0.01, *p* < 0.05) (Fig. 4B, C). In contrast, there was no significant difference in the time and number of entries into the open arms between female *PRRG4*-CKO and *PRRG4*^fl/fl^ mice (Fig. 4B, C).

In the open field test, another anxiety test, the movement trajectories of mice in the open field were recorded to assess anxiety levels (Fig. 4D). Anxious mice have avoidance behavior and do not like to stay in the central area of the open field. The statistical results showed that there was no significant difference in the total distance traveled in the open field between *PRRG4*-CKO and *PRRG4*^fl/fl^ mice, regardless of sex (Fig. 4E), indicating that loss of *PRRG4* did not affect the motor ability of the mice. Importantly, male *PRRG4*-CKO mice spent significantly less time in the central area of the open field (red box) than male *PRRG4*^fl/fl^ mice (*p* < 0.01) (Fig. 4F). In contrast, there was no significant difference in the time spent in the central area of the open field between female *PRRG4*-CKO and *PRRG4*^fl/fl^ mice (Fig. 4F). The results of both tests showed that male *PRRG4*-CKO mice had anxiety symptoms, and the ASD-like behaviors of male *PRRG4*-CKO mice were more pronounced than in female *PRRG4*-CKO mice.

### Pyramidal Neurons Exhibit Abnormal Dendritic Morphology in the Cerebral Cortex and Hippocampus of *PRRG4*-CKO Mice

**Fig. 5:**
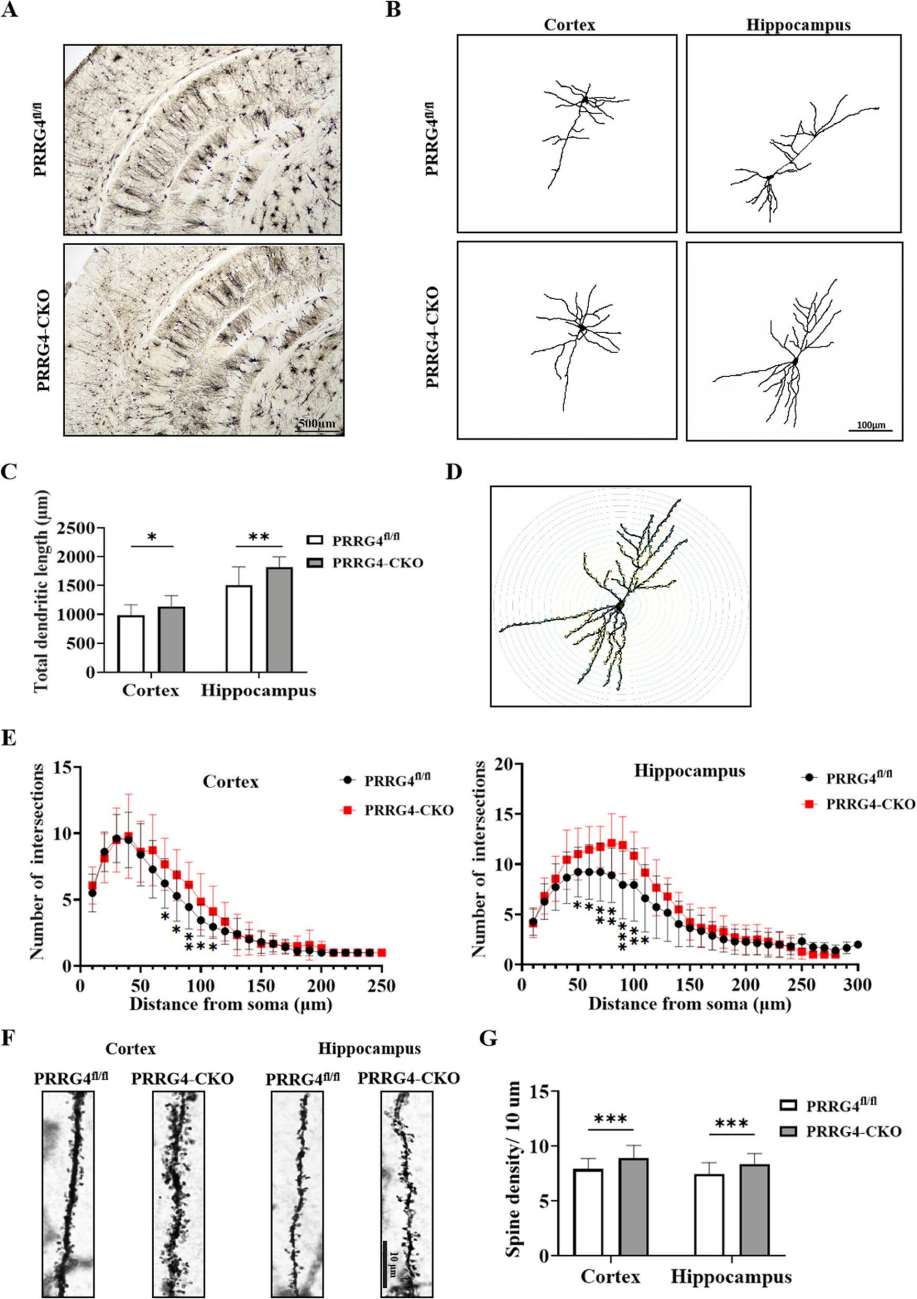
Pyramidal neurons exhibit abnormal dendritic morphology in the cerebral cortex and hippocampus of *PRRG4*-CKO Mice. **A** Microscopic photographs of Golgi-stained mouse brain tissue (scale bar: 500 μm). **B** Representative images of two-dimensional reconstruction of the dendritic trees of pyramidal neurons in cerebral cortex and hippocampus of mice using the Image J plugin (scale bar: 100 μm). **C** Statistical analysis of the average total dendritic length per pyramidal neuron in the cerebral cortex and hippocampus of mice (3 male mice/group, 6 neurons analyzed per mouse). **D** Schematic diagram of analyzing the complexity of pyramidal neuron dendritic branching using the Image J plugin. **E** Statistical analysis of the number of dendritic branches of pyramidal neurons in the cerebral cortex and hippocampus of mice (3 male mice/group, 6 neurons analyzed per mouse). **F** Representative images of dendritic spine density of Golgi-stained pyramidal neurons in the cerebral cortex and hippocampus of mice (scale bar: 10 μm). **G** Statistical analysis of the dendritic spine density of pyramidal neurons in the cerebral cortex and hippocampus of mice (3 male mice/group, 12 neuron dendrites analyzed per mouse). Note: ***: *p* < 0.001, **: *p* < 0.01, *: *p* < 0.05, ns: no significant difference

Considering the important role of neuronal dendritic abnormalities in ASD pathogenesis [13], we wanted to explore whether *PRRG4* knockout affects the dendritic morphology of cerebral cortex and hippocampal neurons. Golgi staining was used to visualize dendritic arborization and dendritic spines of neurons in brain tissues (Fig. 5A). The dendritic trees of pyramidal neurons in cerebral cortex and hippocampus were reconstructed in two dimensions using the Image J plugin, and representative images were shown in Fig. 5B. Statistical analysis of the results showed that *PRRG4*-CKO mice exhibited significantly increased total dendritic length per pyramidal neuron in the cerebral cortex and hippocampus compared to *PRRG4*^fl/fl^ mice (*p* < 0.05, *p* < 0.01) (Fig. 5C). The Image J plugin was also used to analyze the complexity of pyramidal neuron dendritic branching (Fig. 5D). Statistical analysis of the results showed that *PRRG4*-CKO mice displayed a significant increase in the number of dendritic branches of pyramidal neurons, in the cerebral cortex at 70–110 μm from the cell body (*p* < 0.05 or *p* < 0.01) and in the hippocampus at 50–110 μm from the cell body (*p* < 0.05 or *p* < 0.01 or *p* < 0.001), compared to *PRRG4*^fl/fl^ mice (Fig. 5E). In addition, dendritic spine density of Golgi-stained cerebral cortex and hippocampal pyramidal neurons were also analyzed (Fig. 5F). Statistical analysis of the results showed that *PRRG4*-CKO mice showed significantly increased dendritic spine density in cerebral cortex and hippocampal pyramidal neurons compared to *PRRG4*^fl/fl^ mice (*p* < 0.001) (Fig. 5G).

### The Levels of Synaptic Proteins SYP and PSD95 Proteins are Increased in the Cerebral Cortex and Hippocampal Tissues of *PRRG4*-CKO Mice

**Fig. 6:**
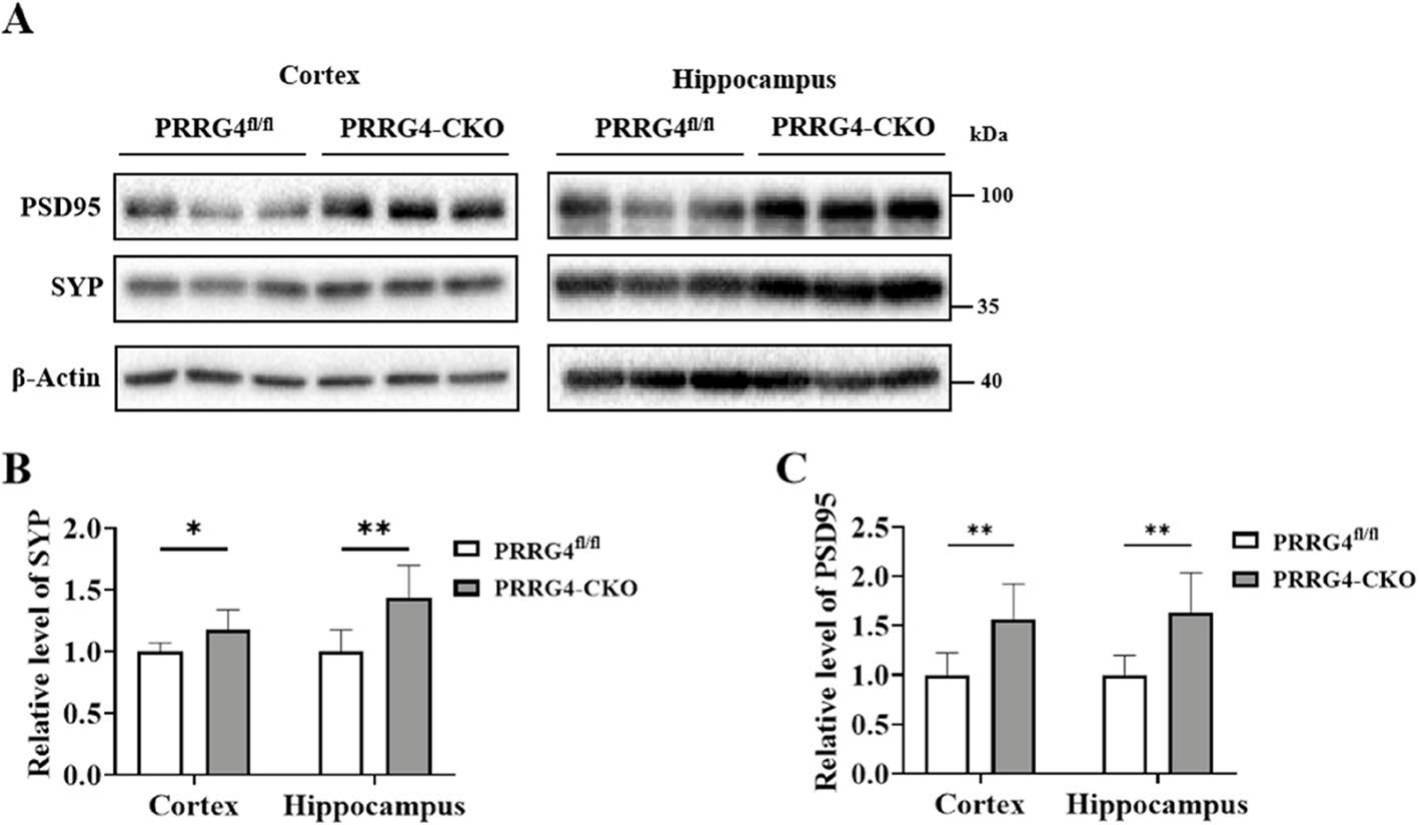
Levels of synaptic proteins SYP and PSD95 are increased in the cerebral cortex and hippocampal tissues of *PRRG4*-CKO mice. **A** Immunoblotting detection of SYP and PSD95 protein levels in the cerebral cortex and hippocampus tissues of mice. **B**, **C** Quantitative statistical analysis of the gray values of SYP and PSD95 protein levels (6 male mice/group). Note: **: *p* < 0.01, *: *p* < 0.05

Presynaptic membrane protein SYP and postsynaptic membrane protein PSD95 play crucial roles in the development and function of neuronal synapses [14, 15]. Immunoblotting was used to detect the levels of presynaptic membrane protein SYP and postsynaptic membrane protein PSD95 in the cerebral cortex and hippocampus tissues of mice (Fig. 6A). The results showed the levels of synaptic proteins SYP and PSD95 were increased in both the cerebral cortex and hippocampus tissues of *PRRG4*-CKO mice compared to *PRRG4*^fl/fl^ mice (*p* < 0.05 or *p* < 0.01) (Fig. 6B, C). Since the increased PSD-95 protein levels often correlate with increased dendritic spine density [16], our data suggest that *PRRG4* knockout may lead to the increased dendritic spine density (Fig. 5F, G) by enhancing PSD95 protein levels.

### PRRG4 Interacts with and MAGI2 in SH-SY5Y Cell and in the Cerebral Cortex and Hippocampal Tissues of *PRRG4*^fl/fl^ Mice

**Fig. 7:**
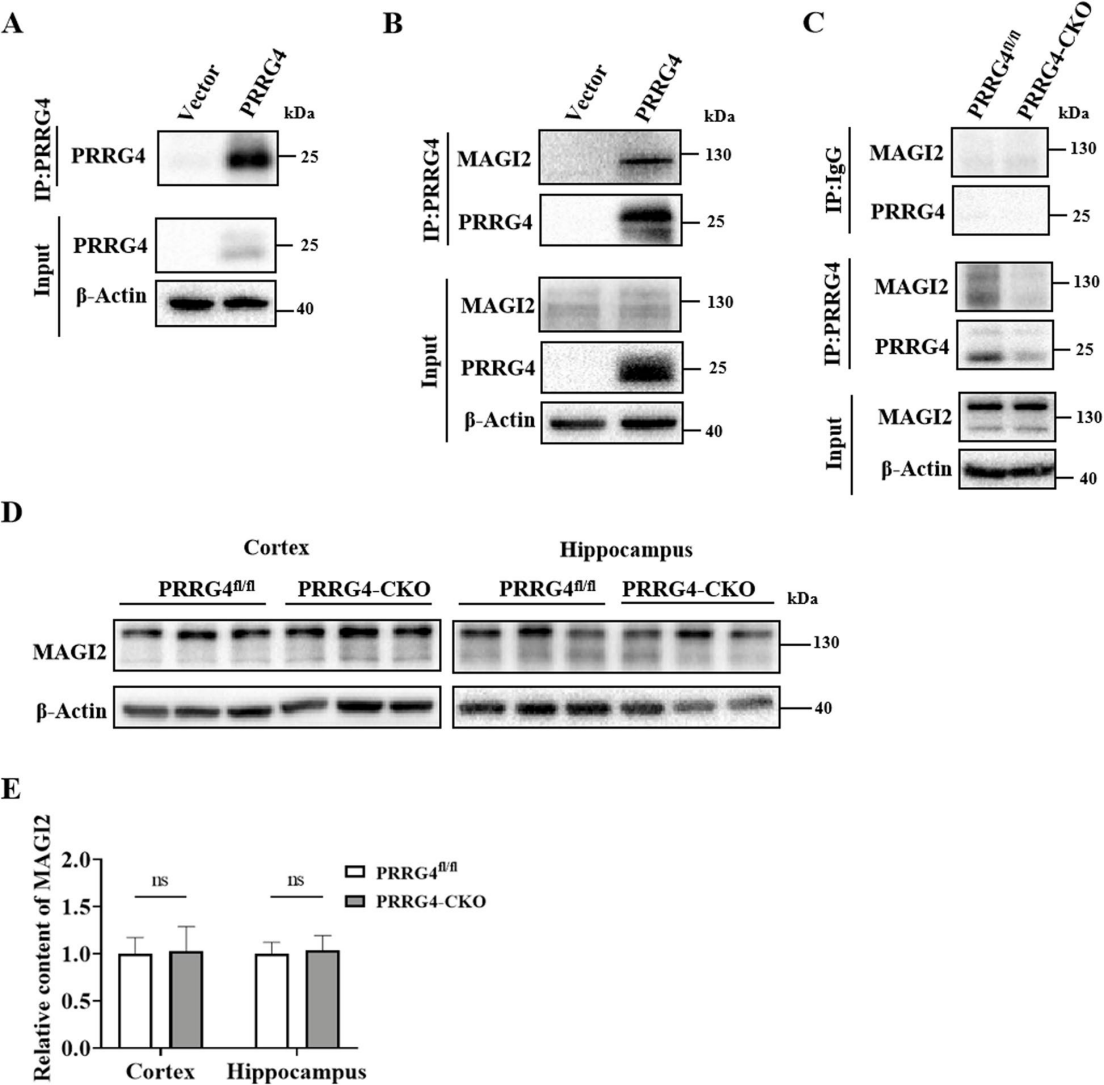
PRRG4 interacts with and MAGI2 in SH-SY5Y cell and in the cerebral cortex and hippocampal tissues of *PRRG4*^fl/fl^ mice. **A** Immunoprecipitation of PRRG4 in SH-SY5Y cells transduced with empty and PRRG4 overexpression vector using purified PRRG4 antibodies. **B** Co-immunoprecipitation detection of the interaction between PRRG4 protein and MAGI2 protein in SH-SY5Y cells. Immunoprecipitation was performed using purified PRRG4 antibodies in SH-SY5Y cells transduced with empty and PRRG4 overexpression vector followed by immunoblotting using PRRG4 and MAGI2 antibodies. **C** Co-immunoprecipitation detection of the interaction between PRRG4 and MAGI2 in mouse brain tissue. Lysates from the cerebral cortex and hippocampus tissues of *PRRG4*^fl/fl^ and *PRRG4*-CKO mice were subjected to immunoprecipitation using purified PRRG4 antibodies or normal rabbit IgG as the negative control, followed immunoblotting using PRRG4 and MAGI2 antibodies. **D** Immunoblotting of MAGI2 content in the cerebral cortex and hippocampus tissues of mice. **E** Quantitative statistical analysis of MAGI2 protein (6 male mice/group). Note: ns: no significant difference

It has been reported that PRRG4 can bind to membrane-associated guanylate kinase 1 (MAGI1) [10]. MAGI2, also a member of the MAGI family proteins, can interact with various synapse-related proteins in neuronal synapses [17]. Therefore, we hypothesized that PRRG4 may regulate synaptic development by interacting with MAGI2. Immunoprecipitation of lysates from SH-SY5Y cells with vector or PRRG4 overexpression using purified PRRG4 antibodies showed that the purified PRRG4 antibodies immunoprecipitated PRRG4 protein (Fig. 7A). In addition, PRRG4 antibodies co-immunoprecipitated MAGI2 (Fig. 7B). More importantly, co-immunoprecipitation experiments showed that PRRG4 and MAGI2 were co-immunoprecipitated in lysates from cerebral cortex and hippocampus tissues of *PRRG4*^fl/fl^ mice, whereas the co-immunoprecipitation of PRRG4 and MAGI2 was very low in brain tissue samples of *PRRG4*-CKO mice (Fig. 7C). There was no significant difference in MAGI2 protein content in the cerebral cortex and hippocampus tissues between *PRRG4*-CKO mice and *PRRG4*^fl/fl^ mice (Fig. 7D, E).

### The Level of Activated RhoA is Decreased in the Cerebral Cortex and Hippocampus Tissues of *PRRG4*-CKO Mice

**Fig. 8:**
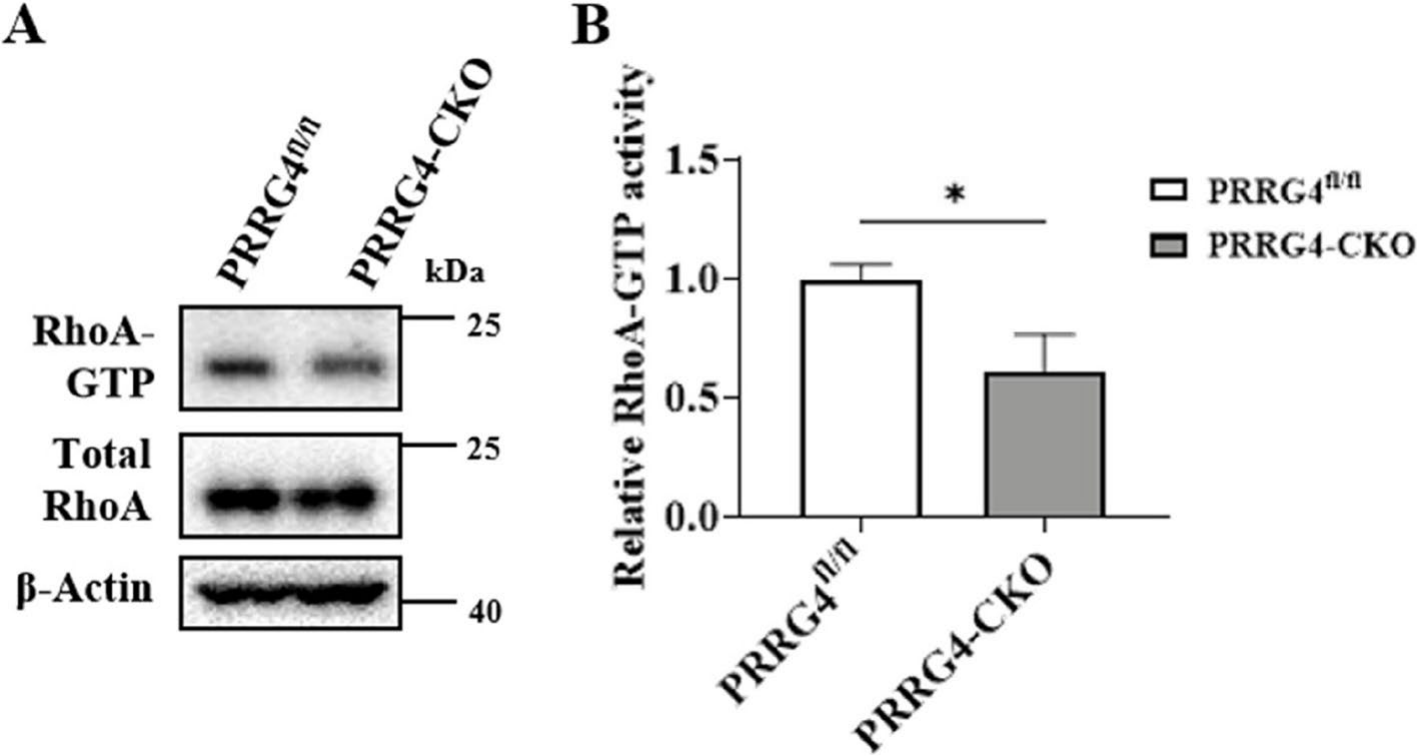
The level of activated RhoA is decreased in the cerebral cortex and hippocampus tissues of *PRRG4*-CKO mice. **A** GST-RBD pull-down assay was used to detect the RhoA-GTP level in the lysates of the cerebral cortex and hippocampus tissues. Then, immunoblotting was performed to detect the levels of RhoA-GTP and total RhoA protein, respectively. **B** Statistical analysis of the levels of RhoA-GTP in total RhoA (3 male mice/group). Note: *: *p* < 0.05

It has been reported that knockout of MAGI2 leads to a decrease in the content of activated RhoA (RhoA-GTP) [18]. Considering that PRRG4 interacted with MAGI2 (Fig. 7C), we hypothesized that knockout of *PRRG4* may affect RhoA activation levels. To verify our hypothesis, we used the GST-RBD pull-down assay to detect RhoA-GTP levels in the cerebral cortex and hippocampus tissues of mice. RBD (Rho-binding domain of Rhotekin) can specifically bind to RhoA-GTP. The results showed that the relative content of RhoA-GTP in the brain tissues of *PRRG4*-CKO mice was approximately 40% lower than that in the brain tissues of *PRRG4*^fl/fl^ mice (*p* < 0.05) (Fig. 8A, B), indicating that knockout of *PRRG4* decreased RhoA activation.

## Discussion

Although the etiology of ASD is not fully understood, increasing evidence suggests that genetic factors play a key role in the development of ASD [19]. ASD risk genes mainly encode proteins related to neuronal synaptic function, gene transcription, and chromatin remodeling [20]. AutDB (http://www.mindspec.org/autdb.html) or named SFARI (Simons Foundation Autism Research Initiative) (https://gene.sfari.org) is real-time updated autism databases [21]. As of October 9, 2024, there were 1109 ASD-related genes (genes with rare single-gene mutations previously reported to be associated with ASD), of which 234 genes with the highest score, and 17 ASD-related copy number variation CNV loci (such as 15q11-q13 duplication, 16p11.2 deletion or duplication) had been recorded. The deletion of the *PRRG4* gene in the current study has not been recorded in the SFARI Gene database.

**Fig. 9:**
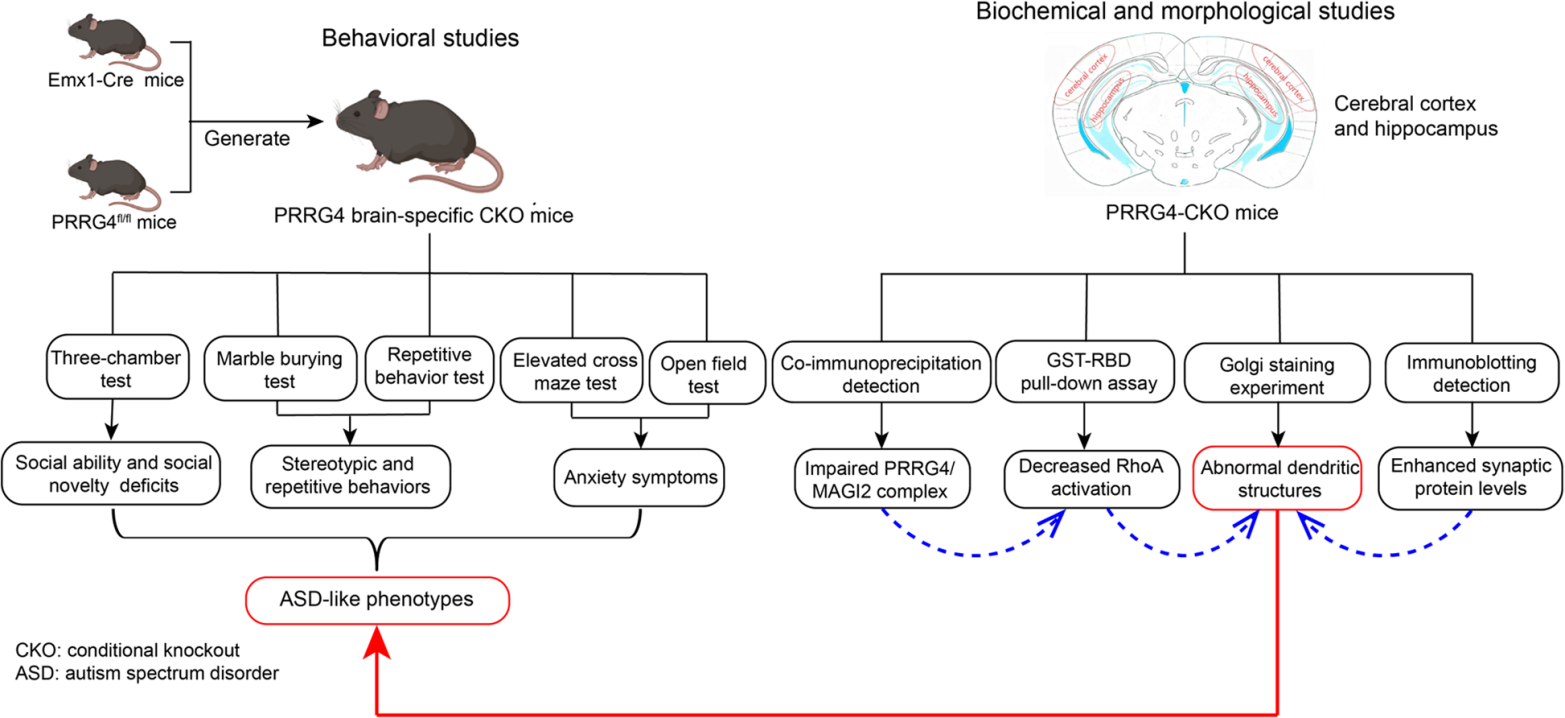
A schematic diagram of the experimental design and conclusions of this study

ASD gene-edited mouse models typically exhibit core ASD symptoms, such as social deficits and repetitive behaviors [22, 23]. As of October 9, 2024, the SFARI Gene database has included ASD gene-edited mouse models of 102 high-risk genes, 131 medium-risk genes, 16 low-risk genes, and 6 CNV loci. The mouse model with *PRRG4* knockout in our study has not been reported so far. Our results showed that brain-specific conditional knockout of *PRRG4* in mice led to ASD-like symptoms (Figs. 2, 3, 4, 9). However, the social novelty deficits and the anxiety symptoms of male *PRRG4*-CKO mice were more pronounced than in female *PRRG4*-CKO mice (Figs. 2, 4). This is similar to the gender difference observed in ASD patients. ASD exhibits a notable sex difference with a 4:1 male to female prevalence ratio with largely unknown mechanistic understanding [2, 24]. In the partially deleted *SHANK3* mouse model, the ASD like symptoms were more pronounced in male mice than in females [25]. In the UBE3A-overexpressing mouse model of ASD, it was reported that male mice exhibited greater impairments in social communication compared to females [24], which was attributed to the defective androgen signaling in the male mice [24]. Likewise, the *CRMP4* knockout mice displayed ASD characteristics, which were more severe in male mice than in females [26]. The mRNA levels of some genes related to neurotransmission and cell adhesion were altered in the brain of *CRMP4* knockout mice, mostly in a gender-dependent manner [26]. Future studies are needed to elucidate the mechanism by which *PRRG4*-CKO mice display sexually dimorphic ASD phenotypes.

The pathogenic mechanism of ASD is mainly related to neuronal synapse and circuit abnormalities during cerebral cortex development. Studies have revealed the importance of cerebral cortex developmental abnormalities in ASD. The frontal cortex of the brain controls higher cognitive processes, such as decision-making, planning, learning, memory, emotion, social behavior, and communication [27]. Due to social and emotional impairments in ASD patients, the frontal cortex has been a region of great interest in recent years [27]. Magnetic resonance imaging studies have shown that the brains of autistic children exhibit abnormal growth patterns, with premature and excessive growth of the frontal cortex [28]. Therefore, our study generated the *PRRG4* brain-specific knockout mice using *Emx1-Cre*. Studies have reported that the *Emx1* promoter specifically drives *Cre* expression in the cerebral cortex and hippocampus of mice [12].

Changes in dendritic complexity and dendritic spine density are accompanied by the formation, maintenance, and disappearance of synapses, thereby regulating the establishment and remodeling of connections within neuronal circuits [29]. Many studies have shown that dendritic and synaptic developmental abnormalities are associated with ASD. Abnormal dendritic spine morphology has been observed in ASD patients and transgenic mice [30]. In addition, increasing evidence suggests that ASD-related genes play a role in synaptic function and neural circuits by directly encoding synaptic scaffolding proteins [31, 32], neurotransmitter receptors, cell adhesion molecules [33], and cytoskeleton-related proteins [3, 34–36]. Knockout of *NLGN3* leads to increased dendritic length and dendritic complexity of pyramidal neurons in the cerebral cortex of mice [37]. In addition, increased dendritic spine density of temporal lobe pyramidal neurons has been found in postmortem ASD patients [38]. In this study, Golgi staining showed that *PRRG4* gene knockout mice had significantly increased total dendritic length, dendritic branching number, and dendritic spine density of cerebral cortex and hippocampal pyramidal neurons (Fig. 5). In addition, immunoblotting detection showed significantly increased levels of synaptic proteins SYP and PSD95 (Fig. 6). Our results suggest that knockout of *PRRG4* may cause ASD symptoms by disrupting dendritic and synaptic development in mice (Fig. 9).

The membrane-associated guanylate kinase family consists of three members, namely MAGI1, MAGI2, and MAGI3. They contain one guanylate kinase (GK) domain, one WW domain, and six PDZ domains in the N-terminal region, which can interact with various proteins with these domain binding motifs [17]. It has been reported that MAGI1 protein can bind to the WW domain binding motif (PY motif) of PRRG4 protein through its WW domain [10], but the biological function of their interaction is unclear. Our data showed that PRRG4 and MAGI2 were co-immunoprecipitated in SH-SY5Y cells overexpressing PRRG4 and in the brain of *PRRG4*^fl/fl^ mice, indicating that MAGI2 interacts with PRRG4 (Fig. 7B, C). Theoretically, MAGI2 via its WW domain can interact the PY motif in PRRG4.

The relationship between MAGI2 and ASD has not been reported. MAGI2, which is more widely expressed in neurons, is known as a synaptic scaffolding molecule [39]. It plays a key role in synapses by interacting with various cell adhesion molecules, receptors, and signaling molecules (such as TARP and NLGN3 proteins) on the postsynaptic membrane [17, 40]. MAGI2 can regulate the number of AMPA receptors on synapses by interacting with TARP, thereby affecting the strength of excitatory synaptic transmission [41]. NLGN3 can recruit MAGI2 to the cell membrane region, and then MAGI2 stabilizes PTEN expression by binding to PTEN, further inhibiting the AKT/mTOR signaling pathway, thereby inhibiting excessive dendrite formation [37]. However, NLGN3 could not be detected in PRRG4 immunoprecipitates from mice brain tissue (data not shown). Moreover, mTOR signaling was not affected in *PRRG4*-CKO mice compared to P*RRG4*^*fl/fl*^ mice (data not shown). Our study suggests that disruption of the MAGI2/PRRG4 interaction may contribute to ASD independent of NLGN3.

RhoA protein can regulate neuronal dendrite, axon, and synapse development and has been identified to be associated with ASD [42, 43]. In neuronal cells, one of the main functions of the Rho GTPases is to regulate the assembly and organization of the actin-myosin cytoskeleton, which plays an important regulatory role in neuronal growth cone dynamics, dendritic spine formation, and axon extension and guidance [44]. Many neuronal studies support the general model in which Rac and Cdc42 stimulate these processes, whereas RhoA inhibits these processes, leading to axon and dendrite retraction, and dendritic spine and synapse loss [44]. Rho GTPase signaling dysfunction accounts for a considerable proportion of ASD pathogenesis. Twenty genes encoding regulators and effectors of Rho GTPases (mainly including RhoA) are listed as ASD risk genes in the SFARI database [43]. Studies have shown that RhoA-GTP can reduce the extension of neuronal dendritic branches and dendritic spine density. Conversely, reducing RhoA-GTP content leads to increased extension of dendritic branches [42]. Our data showed that *PRRG4*-CKO mice exhibited ASD-like symptoms (Figs. 2, 3, 4), decreased levels of RhoA-GTP in the cerebral cortex and hippocampus (Fig. 8), abnormally increased total dendritic length, dendritic branching number, and dendritic spine density of pyramidal neurons, and abnormally increased synaptic protein levels (Figs. 5, 6). Therefore, the results of our study strongly suggest that decreased RhoA activation contributes to the dendritic and synaptic developmental abnormalities and ASD symptoms in the *PRRG4* knockout mice (Fig. 9).

Studies have shown that MAGI2 can stabilize RhoA activity and further regulate RhoA signaling [45]. Iida et al. found that MAGI2/S-SCAM is a scaffold required for dendritic response to NMDA receptor signaling to activate RhoA protein. MAGI2 mediates NMDA receptor signaling to activate RhoA, thereby regulating dendritic spine plasticity [18]. Decreased RhoA-GTP levels and increased elongated dendritic spines were found in primary cultured neurons from MAGI2 knockout mice [18]. The results of this study showed that PRRG4 interacted with MAGI2 (Fig. 7B, C), and RhoA-GTP levels were reduced in the cerebral cortex and hippocampus of *PRRG4*-CKO mice (Fig. 8). We speculated that PRRG4 promotes RhoA activation through its interaction with MAGI2, thereby regulating dendrite and dendritic spine homeostasis.

In conclusion, our study suggests that PRRG4 protein may affect mouse dendrite and synapse development by activating RhoA through interaction with MAGI2. Knockout of *PRRG4* may cause ASD due to the disruption of the PRRG4/MAGI2/RhoA pathway (Fig. 9). Our findings support the association between *PRRG4* loss and ASD phenotypes observed in WAGR syndrome.

## Data Availability

No datasets were generated or analysed during the current study.

## Abbreviations

ASD: Autism Spectrum Disorder
CKO: Conditional Knockout
DAPI: 4’,6-Diamidino-2-Phenylindole
ECL: Enhanced Chemiluminescence
GST: Glutathione-S-Transferase
MAGI2/S-SCAM: Membrane-Associated Guanylate Kinase Inverted-2/Synaptic Scaffolding Molecular
PAGE: Polyacrylamide Gel Electrophoresis
PBS: Phosphate Buffer Saline
PRRG4: Proline-Rich Gla Domain 4
PVDF: Polyvinylidene Fluoride
qPCR: Quantitative PCR
RBD: Rho-Binding Domain
RhoA: Ras Homolog Gene Family Member A
SDS: Sodium Dodecyl Sulfate
WAGR: (Wilms’ Tumor, Aniridia, Gonadoblastoma, Mental Retardation)
